# The use of a viral 2A sequence for the simultaneous over-expression of both the *vgf* gene and enhanced green fluorescent protein (eGFP) *in vitro* and *in vivo*

**DOI:** 10.1016/j.jneumeth.2015.08.013

**Published:** 2015-12-30

**Authors:** Jo E. Lewis, John M. Brameld, Phil Hill, Perry Barrett, Francis J.P. Ebling, Preeti H. Jethwa

**Affiliations:** a Division of Nutritional Sciences, School of Biosciences, University of Nottingham, Sutton Bonington Campus, Loughborough LE12 5RD, UK; b School of Life Sciences, University of Nottingham Medical School, Queen's Medical Centre, Nottingham NG7 2UH, UK; c The Rowett Institute of Nutrition and Health, University of Aberdeen, Bucksburn, Aberdeen AB21 9SB, UK

**Keywords:** Viral 2A sequence, Recombinant adeno-associated virus (rAAV), Neuroblastoma SH-SY5Y cells, VGF (non-acronymic), Enhanced green fluorescent protein (eGFP), Siberian hamster

## Abstract

**Introduction:**

The viral 2A sequence has become an attractive alternative to the traditional internal ribosomal entry site (IRES) for simultaneous over-expression of two genes and in combination with recombinant adeno-associated viruses (rAAV) has been used to manipulate gene expression *in vitro*.

**New method:**

To develop a rAAV construct in combination with the viral 2A sequence to allow long-term over-expression of the *vgf* gene and fluorescent marker gene for tracking of the transfected neurones *in vivo*.

**Results:**

Transient transfection of the AAV plasmid containing the *vgf* gene, viral 2A sequence and eGFP into SH-SY5Y cells resulted in eGFP fluorescence comparable to a commercially available reporter construct. This increase in fluorescent cells was accompanied by an increase in VGF mRNA expression. Infusion of the rAAV vector containing the *vgf* gene, viral 2A sequence and eGFP resulted in eGFP fluorescence in the hypothalamus of both mice and Siberian hamsters, 32 weeks post infusion. *In situ* hybridisation confirmed that the location of VGF mRNA expression in the hypothalamus corresponded to the eGFP pattern of fluorescence.

**Comparison with old method:**

The viral 2A sequence is much smaller than the traditional IRES and therefore allowed over-expression of the *vgf* gene with fluorescent tracking without compromising viral capacity.

**Conclusion:**

The use of the viral 2A sequence in the AAV plasmid allowed the simultaneous expression of both genes *in vitro*. When used in combination with rAAV it resulted in long-term over-expression of both genes at equivalent locations in the hypothalamus of both Siberian hamsters and mice, without any adverse effects.

## Highlights

• The viral 2A sequence is suitable for gene manipulation in the Siberian hamster.
• It allows long-term simultaneous over-expression of 2 genes *in vitro* and *in vivo*.
• We demonstrate dual expression *in vitro* in the neuroblastoma cell line SH-SY5Y.
• We demonstrate dual expression in the hypothalami of Siberian hamsters and mice.

**Abbreviations**: All standard in this field

## 1 Introduction

The use of transgenic and knockout mice has contributed greatly to our understanding of gene function and disease. However, these mice models are not only complex to create, but may not be viable, or may produce a complex phenotype reflecting developmental or functional compensation. Gene transfer technologies utilising viruses provide a means to overcome some of these limitations. The most widely used viral vectors for neuronal over-expression are recombinant adeno-associated viruses (rAAV), as they have been shown to efficiently and stably transduce a variety of tissues in immunocompetent animals, including mice, rats and Siberian hamsters (Daya and Berns, 2008, Jethwa et al., 2010). However these viruses are limited in their capacity to incorporate foreign DNA by virtue of their genomic size (approximately 5 kb) (Kay et al., 2001). Although hybrids with other viruses have been generated to try to overcome this limitation (Costantini et al., 1999, Nakai et al., 2000, Recchia et al., 1999), the low insert capacity remains an issue if co-expression of genes is required from a single viral vector. For example, the combination of a gene of interest and a reporter gene, such as green fluorescent protein (GFP), is particularly useful in order to enable visualisation of transduced cells both *in vitro* and *in vivo*.

Co-expression of genes can greatly enhance the efficiency and usefulness of transgenic applications. A number of options are available to simultaneously express two genes in particular cells/neurones. Currently the most common approach relies on vector strategies in which genes are linked by an internal ribosomal entry site (IRES) to allow simultaneous expression of genes, since the IRES sequence permits the production of multiple proteins from a single mRNA transcript. Ribosomes bind to the IRES in a 5′-cap independent manner and initiate translation (Jang et al., 1988). However, there are two main limitations to the use of the IRES. First, the size of the IRES is often in excess of 500 base pairs, which further limits the capacity to incorporate foreign DNA. Second, the expression of the downstream gene within the vector is often reduced and therefore expression of the two genes is not equivalent. Recently it has been shown (Chan et al., 2011) that viral 2A sequences can overcome these limitations; with the viral 2A sequence shown to increase reporter gene expression in comparison to the IRES sequence. Thus the viral 2A peptide sequence offers an alternative to the IRES.

The viral 2A sequence was first identified in the picornaviruses (Ryan et al., 1991, Trichas et al., 2008). Viral 2A sequences are relatively short (approximately 20 amino acids) and contain the consensus motif Asp-Val/Ile-Glu-X-Asn-Pro-Gly-Pro (Ibrahimi et al., 2009). The sequence acts co-translationally, the formation of a normal peptide bond between the glycine and proline residues is prevented, which results in ribosomal skipping (Donnelly et al., 2001) and therefore cleavage of the nascent polypeptide. This effect produces multiple genes at equimolar levels. The efficiency of the viral 2A sequence has been demonstrated in a wide range of eukaryotic cells from yeast to mammals (de Felipe and Ryan, 2004), including its effectiveness following insertion between two reporter genes *in vitro* (Ryan and Drew, 1994). Importantly, the viral 2A sequence, in combination with AAV, was able to function in the rat brain without any cytotoxic effects (Furler et al., 2001).

Despite these advantages, the use of viral 2A sequence technology for *in vivo* research has been limited. In this study, we utilised the viral 2A sequence (a) to overcome AAV capacity problems faced utilising large genes and (b) to over-express VGF and eGFP in the hypothalamus of Siberian hamsters and mice to determine its feasibility in standard and non-standard laboratory animals. We clearly demonstrate that the combination of AAV with the viral 2A sequence results in long term over-expression of VGF mRNA and the eGFP reporter gene in the hypothalamus of both species.

## 2 Materials and methods

### 2.1 Synthesis of construct and viral particles

**Fig. 1:**
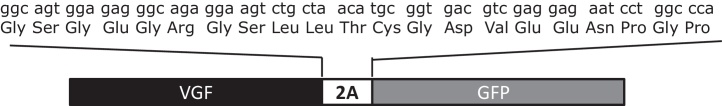
*The viral 2A sequence*. The viral 2A sequence, containing the consensus motif Asp-Val-Glu-X-Asn-Pro-Gly-Pro, which mediates the co-translational cleavage of the nascent polypeptide, resulting in expression of both the VGF gene and eGFP reporter gene in equimolar amounts. Adapted from Trichas et al. (2008).

Construction of the pAAV-CBA-AgRP-IRES-eGFP-WPRE plasmid (from an original plasmid AAV vector, a kind gift from Dr Miguel Sena Esteves, University of Massachusetts, Worcester, USA) has been described previously (Jethwa et al., 2010). We replaced the AgRP-IRES in this plasmid with VGF cDNA and the viral 2A sequence from Trichas et al. (2008) (Fig. 1). Full length mouse VGF cDNA (Accession no.: NM_001039385.1) was isolated and inserted into the blunt cloning vector, pSC-B-AMP/KAN (Agilent Technologies, UK) for amplification using modified PCR primers. The forward primer contained a Kozak sequence for initiation of translation, while the reverse primer contained the viral 2A sequence and a point mutation to allow the removal of the stop codon from the VGF cDNA (Table 1).

**Fig. 2:**
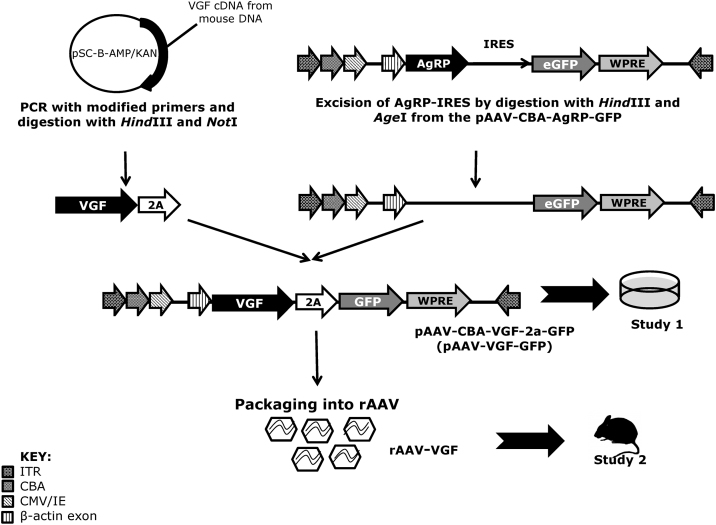
*A schematic to show steps leading to the production of rAAV-VGF*. Full length mouse cDNA was isolated and inserted into the blunt cloning vector pSC-B-AMP/KAN for amplification using modified primers to produce the VGF-2A fragment. This product was cloned into the pAAV-CBA-AgRP-IRES-GFP backbone (previously used in Jethwa et al., 2010), following excision of the AgRP-IRES complex to produce pAAV-CBA-VGF-2a-GFP (pAAV-VGF-GFP). This was used for the *in vitro* checking (Study 1), before then being used to produce recombinant AAV-2 (rAAV) by Vector BioLabs (USA) yielding rAAV-VGF for *in vivo* infusion (study 2). ITR—internal tandem repeat, CMV/IE—cytomegalovirus intermediate early promoter, CBA—chicken β-actin promoter, eGFP—enhanced green fluorescent protein, WPRE—Woodchuck Hepatitis virus post-transcriptional regulatory element.

The resulting PCR fragment, which contained the VGF cDNA and viral 2A sequence (VGF-2A), was digested with *Hind*III and *Not*I, while the AgRP-IRES sequence was excised from the pAAV-CBA-AgRP-IRES-eGFP-WPRE plasmid vector using *Hind*III and *Age*I, the latter producing an identical 5′ overhang (5′-GGCC-3′) to *Not*I. The digested plasmid was treated with calf intestinal alkaline phosphatase (Promega, USA) to prevent re-ligation of the plasmid without insert. Sure 2 Supercompetent cells (Agilent Technologies, USA) were used for transformation to prevent DNA rearrangement/deletion, as they lack the *Escherichia coli* genes implicated in such events, thereby improving cloning efficiency. The VGF-2A PCR fragment produced by PCR was cloned in to the original AgRP plasmid to produce the pAAV-CBA-VGF-2A-eGFP-WPRE plasmid vector (referred to as pAAV-VGF-GFP) (Fig. 2).

Following *in vitro* validation in SH-SY5Y cells, the pAAV-VGF-GFP plasmid was packaged into AAV-2 particles by Vector BioLabs (PA, USA). The titre obtained was 7.2 × 10^12^ gc/ml and will be referred to as rAAV-VGF. The rAAV-GFP control vector was purchased from Vector Biolabs (PA, USA); this had a viral titre of 1 × 10^13^ gc/ml and expressed eGFP under the control of a cytomegalovirus (CMV) promoter.

### 2.2 The SH-SY5Y neuroblastoma cell line

The human neuroblastoma SH-SY5Y cells were grown in 25 cm^2^ tissue culture flasks with complete Dulbecco's modified Eagle's medium/nutrient mixture F-12 Ham, containing 10% foetal bovine serum, 100 units/l penicillin and 100 mg/l streptomycin (all Sigma-Aldrich, Dorset, UK), which will be referred to as DMEM/F12 complete. Cultures were maintained at 37 °C in a 95% humidified incubator with 5% CO_2_. Cells were routinely split 1:3 with 0.05% trypsin (Sigma-Aldrich, Dorset, UK) every 48 h. For differentiation, the SH-SY5Y cells were seeded at 5 × 10^4^ cells/cm^2^ in 6-well tissue culture plates (9.5 cm^2^) in DMEM/F12 complete, and 48 h later treated with 10 μM retinoic acid (RA) (Sigma-Aldrich, Dorset, UK) for 120 h in accordance with previous studies (Cheung et al., 2009, Pahlman et al., 1984). Cells were visualised by Leica DF420C microscope (Leica Instruments, Milton Keynes, UK).

### 2.3 Study 1: *In vitro* assessment of viral plasmid pAAV-VGF-GFP

#### 2.3.1 Transfection

pAAV-VGF-GFP and pZsGreen1-N1 (a positive control; Clontech Laboratories, CA, USA) plasmids were transfected into undifferentiated SH-SY5Y cells using FuGENE HD (Promega, Wisconsin, USA) according to the manufacturer's protocol. Briefly, prior to transfection, cells at approximately 80% confluence were transferred to OptiMeM1^®^ reduced serum media containing no antibiotics (Promega, Wisconsin, USA). Plasmid DNA was diluted in OptiMEM1^®^ to give a concentration of 20 μg/ml. The FuGENE^®^ HD transfection reagent was added to achieve a 3:1, reagent: DNA ratio and 10 μl of the reagent:DNA mixture was added to each well equating to 50 ng of plasmid DNA per well. The cells were incubated for 72 h prior to assessment of eGFP fluorescence and VGF mRNA expression. Cells transfected in parallel were further incubated with RA (10 μM) to induce differentiation prior to eGFP assessment.

#### 2.3.2 *In vitro* eGFP expression

eGFP fluorescence in the SH-SY5Y cells was imaged using the Typhoon Trio+ instrument (excitation maximum = 493 nm; emission maximum = 505 nm) and ImageQuant software (GE LifeSciences, UK).

#### 2.3.3 *In vitro* VGF mRNA expression

##### 2.3.3.1 RNA extraction

Prior to harvesting the cells, the DMEM complete medium was removed, then cells were scraped directly into 200 μl of RNase-free phosphate buffered saline (PBS). Total RNA was extracted from the cells using the High Pure Isolation kit according to the manufacturer's instructions (Roche Life Science, West Sussex, UK) and as described previously (Brown et al., 2012).

##### 2.3.3.2 cDNA synthesis and amplification by PCR

First strand complimentary DNA (cDNA) was synthesised using the Transcriptor First Strand cDNA Synthesis kit (Roche Life Science, West Sussex, UK) according to the manufacturer's protocol. Briefly 2 μg of total RNA was combined with 60 μM random hexamer primers and RNase free water and incubated for 10 min at 65 °C before then being placed on ice. Then 0.5 μl transcriptor reverse transcriptase (10 U), 2 μl deoxynucleotide mix (1 mM each), 0.5 μl protector RNase inhibitor (20 U) and 4 μl reaction buffer were added to each reaction on ice, vortexed and incubated for 10 min at 25 °C for 10 min, followed by incubations at 55 °C (30 min), 85 °C (5 min) and then 4 °C to cool. The cDNA was stored at 20 °C.

##### 2.3.3.3 Quantitative RT-PCR

Quantitative Polymerase Chain Reactions (PCR) were performed with SYBR^®^ green optimised for the LightCycler 480 (Roche Life Science, UK). All reactions were performed in triplicate on 384 well plates as per manufacturer's instructions. Briefly each well contained 5 μl cDNA in SYBR^®^ Master Mix 1, 0.25 μM primers, and RNase free water in a total volume of 15 μl per well. Samples were pre-incubated at 95 °C for 5 min, followed by 40 amplification cycles (de-naturation: 95 °C for 10 s; annealing: 55 °C for 10 s and elongation: 72 °C for 30 s). The respective primer sets used can be seen in Table 1.

*C*_*t*_ values were converted to expression levels using a standard curve produced using a serial dilution of a pooled cDNA made from all samples to check linearity and efficiency of the PCR reactions. Expression values were then normalised to cyclophilin A, the most stable reference gene under the experimental conditions.

### 2.4 Study 2: *In vivo* assessment of the rAAV-VGF viral construct

#### 2.4.1 Animals

All animal procedures were approved by the University of Nottingham Local Ethical Review Committee and were carried out in accordance with the UK Animals (Scientific Procedures) Act 1986 (project licence PPL 40/3604).

Male Siberian hamsters (*Phodopus sungorus*) aged 4–6 months (*n* = 12) were taken from a colony maintained by the University of Nottingham (Ebling, 1994). They were housed in individual cages under controlled temperature (21 ± 1 °C) and on a photocycle of 16 h light/8 h dark (lights off at 11:00 h), with *ad libitum* access to food and water, unless otherwise stated. The diet was standard laboratory chow comprising of 19% extruded protein and 9% fat (Teklad 2019, Harlan, UK).

Male C57BL/6 J mice (*Mus musculus*) aged 3 months (*n* = 12), were supplied by Harlan, UK. They were housed in individual cages under controlled temperature (21 ± 1 °C) and on a photocycle of 12 h light/12 h dark (lights off at 19:00), with *ad libitum* access to food and water. The diet was standard laboratory chow comprising of 18% extruded protein and 6.2% fat (Teklad 2018, Harlan, UK).

#### 2.4.2 Infusion of viral vectors

Animal surgical procedures were carried out as previously described (Jethwa et al., 2010). Briefly, animals were placed in a Kopf stereotaxic frame (David Kopf Instruments, NY, USA) under general anaesthesia (0.5–2.5% isoflurane), with the incisor bar positioned level with the interaural line. Using the sutures confluence bregma as a landmark, a small hole was drilled on midline. The dura mater was pierced just lateral to the mid-sagittal sinus and a drawn glass capillary microinjector (30-micron tip diameter) was lowered to the correct location. Using a Nanolitre Injection system (WPI, Stevenage, UK), Siberian hamsters (*n* = 6/group) or mice (*n* = 6/group) were bilaterally infused with 200 nl of the viral vector (rAAV-GFP or rAAV-VGF-GFP) directed towards the PVN (anteroposterior +0.03, mediolateral ±0.03, dorsoventral −0.58 (co-ordinates from Paxinos and Franklin, 2012) over one minute. Following infusion, the glass microinjector was kept in place for an additional 5 min to allow for diffusion and prevention of backflow through the cannula track, the incision was closed using Michel clips. Analgesia was maintained *via* subcutaneous injection of carprofen (50 mg/kg Rimadyl, Pfizer, Kent, UK) administered before surgery. The surgically-prepared Siberian hamsters and mice were allowed a seven day recovery period, during which they were handled on a daily basis, received analgesia and had access to a palatable diet consisting of soaked Teklab diet. One week post viral infusion, Siberian hamsters and mice underwent metabolic analysis to determine locomotor activity in the Comprehensive Laboratory Animal Monitoring System (CLAMS) as previously described (Jethwa et al., 2010). At 32 weeks post infusion, animals were euthanised by injection of pentobarbital sodium (Euthatal; Rhone Merieux, Harlow, UK). Brains were immediately removed and snap frozen on dry ice for subsequent measurement of GFP and VGF expression by fluorescence imaging and *in situ* hybridisation, respectively.

#### 2.4.3 *In situ* hybridisation

Coronal sections (20 μm) of brains were cut, using a cryostat (Microm, USA), and arranged on 3-aminopropyltriethoxysaline (APES) coated slides. Sections on the slides were fixed in 4% paraformaldehyde (PFA) before being dried for storage at −70 °C. Riboprobes were generated from the blunt pSC-B-AMP/KAN plasmid (containing VGF cDNA) and pSLAX13-eGFP (containing eGFP—a kind gift from Dr Dylan Sweetman, University of Nottingham). Antisense transcripts were generated using T7 polymerase (NEB, USA) in the presence of digoxigenin (DIG)/fluorescein-12-uridine-5-triphosphate, which could subsequently be detected. Fluorescence *in situ* hybridisation was performed as described previously (Sweetman et al., 2008). Briefly, riboprobes were purified on a G-50 spin column (Boehringer Mannheim, USA) and a small aliquot subjected to gel electrophoresis to determine yield and integrity of the riboprobes. Slides containing the sections were thawed to room temperature, fixed with freshly prepared 4% PFA/0.1% gluteraldehyde before being treated with proteinase K (10 μg/ml). Slides were incubated with 80 μl 1× hybridisation solution (Sigma, St. Louis, MO, USA) containing 1 μl of riboprobe for 6 h at 65 °C/min at room temperature. A cover slip was placed on each slide and slides were incubated overnight at 65 °C in a humidified *in situ* hybridisation chamber. Post hybridisation, the section were washed twice with hybridisation solution at 65 °C for 10 min, followed by 2 washes with maleic acid buffer containing 0.1% Tween-20 (MABT, pH 7.5) at room temperature. The sections were blocked with MABT/2%Roche blocking agent (Roche, USA) for 1 h at room temperature and incubated overnight with anti-digoxigenin antibody conjugated to alkaline phosphatase (1:2000) at 4 °C. Slides were subsequently washed 3× with MABT for 1 h at room temperature following an overnight incubation at 4 °C in MABT. For the colour reaction, sections were washed with 1-methyl-5-thiotetrazole (NMTT) containing nitroblue tetrazolium (NBT) and 5-bromo-3-indoxyl-phosphate (BCIP), which yields a black–purple precipitation. The reaction was stopped by washing the sections in 5× TBSTw (1×TBS, 0.1% Tween™ 20, 0.2 mM sodium azide) overnight at 4 °C. This process was repeated the following day to intensify the signal and reduce background. Cover slips were applied to the slides with a small amount of Vectashield media (Vector Labs, CA, USA) and sealed with nail polish. Images were captured using a Leica DMRB microscope (Leica Microsystems, Germany) and Openlab software (UK). eGFP was excited using the 488 nm laser (emission 520 nm).

### 2.5 Statistical analysis

Descriptive statistics (means and standard error mean (SEM)) were generated using GraphPad PRISM version 6.0 (GraphPad Software Inc, USA). Transfection studies (Section 2.3) and food intake data (Section 2.4) were compared using a one-way ANOVA. A two-way ANOVA (treatment *vs.* time) was used to analyse the locomotor activity data from CLAMS (Section 2.4). *P* < 0.05 was considered statistically significant.

## 3 Results

### 3.1 Study 1: *In vitro* assessment of viral plasmid pAAV-VGF-GFP

**Fig. 3:**
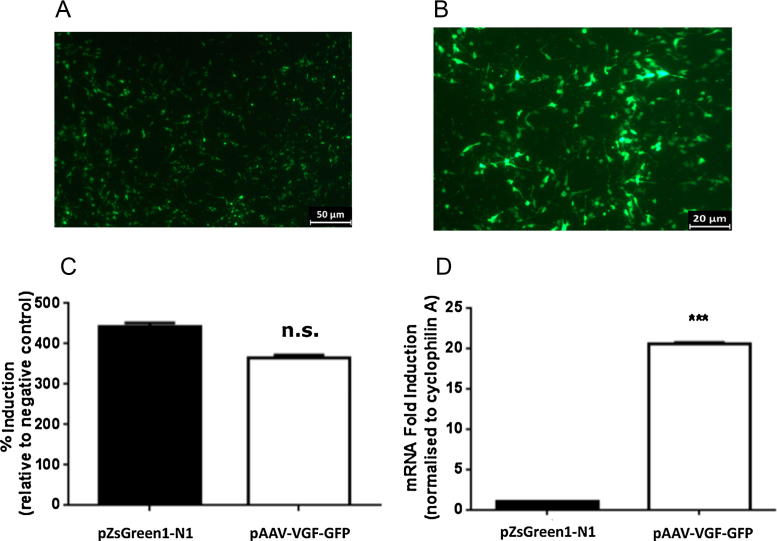
*eGFP and VGF expression in SH-SY5Y cells following transient transfection with the pAAV-VGF-GFP plasmid*. (A) eGFP expression in SH-SY5Y cells 72 h post transfection with the pAAV-VGF-GFP plasmid. (B) eGFP expression in the SH-SY5Y cells transfected with the pAAV-VGF-GFP plasmid following differentiation with 10 μM RA for 120 h. Quantification of (C) eGFP expression and (D) VGF mRNA 72 h post transfection with pAAV-VGF-GFP or pZsGreen1-N1 (control) in undifferentiated SH-SY5Y cells. Values are group means ± SEM (*n* = 3), *** *p* < 0.0001 *vs.* control.

Prior to viral packaging, the ability of the pAAV-VGF-GFP plasmid to simultaneously express both VGF and eGFP was verified following transient transfection of SH-SY5Y cells. Fluorescent microscopy confirmed widespread expression of eGFP in undifferentiated SH-SY5Y cells 72 h post transfection (Fig. 3A). Treatment of the transfected SH-SY5Y cells with 10 μM RA for 120 h induced differentiation, at which point eGFP expression expanded beyond the cell body and extended to the neurite projections (see Fig. 3B). The increase in eGFP expression in undifferentiated cells was similar to that observed following transfection of the SH-SY5Y cell line with the positive control construct, pZsGreen1-N1, expressing ZsGreen1-N1 under the control of a CMV promoter (GFP expression: pZsGreen1-N1; 442.8 ± 8.8, pAAV-VGF-GFP; 366.0 ± 6.5, *p* = n.s. Fig. 3C). Furthermore, the increase in eGFP expression in undifferentiated cells was associated with an increase in VGF mRNA expression. Transfection of the SH-SY5Y cells with pAAV-VGF-GFP resulted in a 20-fold increase in VGF mRNA expression (VGF mRNA: 20.6 ± 0.1, *F* = 5.830, *p* < 0.0001, Fig. 3D), compared to control cells transfected with pZsGreen1-N1.

### 3.2 Study 2: *In vivo* assessment of viral construct rAAV-VGF

#### 3.2.1 Animal behaviour

Approximately 95% of the animals that underwent the viral microinjection surgery recovered fully from anaesthesia and were reintroduced to their home cages. Once reintroduced, all animals maintained good health throughout the duration of the study and normal behaviour was observed, with no changes in initial food intake or locomotor activity observed one week post viral infusion (Table 2).

#### 3.2.2 Hypothalamic expression of eGFP and VGF mRNA

**Fig. 4:**
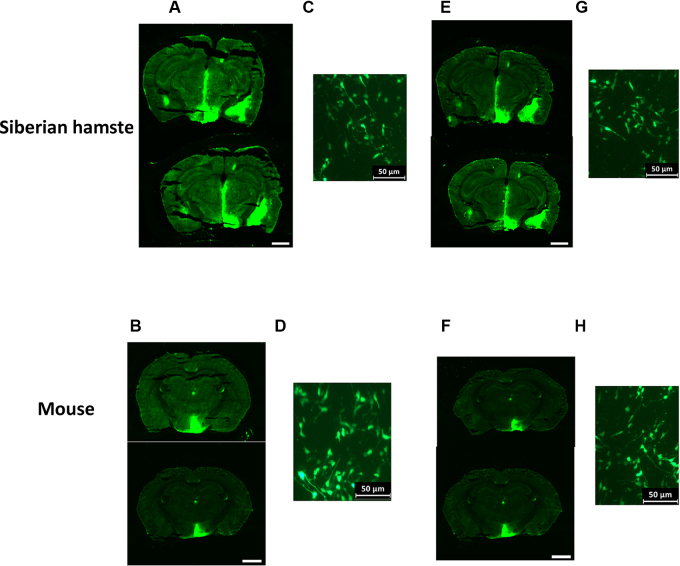
*Use of the viral 2A sequence in the rAAV-VGF construct results in widespread eGFP expression in the hypothalamus*. Representative pictures of eGFP expression in the hypothalamus of Siberian hamsters and mice which received bilateral (200 nl) injections of either rAAV-GFP or rAAV-VGF directed to the PVN. (A–D) eGFP expression following infusion of rAAV-GFP control vector in the whole hypothalamus of (A) Siberian hamsters and (B) mice and in the cell soma and processes of the PVN of (C) Siberian hamsters and (D) mice. (E–G) eGFP expression following infusion of the rAAV-VGF vector in the hypothalamus of (E) Siberian hamsters and (F) mice and in the cell soma and processes of the PVN of (G) Siberian hamsters and (H) mice. Scale bar = 2.5 mm.

Post mortem analysis of eGFP by fluorescence microscopy was performed at 32 weeks post infusion of the viral constructs rAAV-GFP and rAAV-VGF in Siberian hamsters and mice. Expression of eGFP was widespread in the hypothalamus of both species, as well as along the infusion tract. eGFP expression was observed in both rAAV-GFP and rAAV-VGF infused Siberian hamsters and mice. The spread of the eGFP expression from viral constructs was similar for both rAAV-GFP and rAAV-VGF vectors, up to 400 μm in the lateral plane (Fig. 4). As seen in previous studies (Jethwa et al., 2010), there were instances of unilateral expression in several of the animals, which was independent of the vector infused (Fig. 4).

Within the hypothalamus, eGFP expression was apparent along the third ventricle, suprachiasmatic nucleus, lateral hypothalamic area, the anterior hypothalamic nucleus, the paraventricular nucleus (PVN), arcuate nucleus (ARC), ventromedial hypothalamus and dorsomedial nucleus in both species receiving either rAAV-GFP or rAAV-VGF viral vectors (Fig. 4). Confocal analysis revealed there was widespread eGFP expression in the cell soma and cell processes of the PVN of both Siberian hamsters (Fig. 4C and G) and mice (Fig. 4D and H) receiving either rAAV-GFP or rAAV-VGF viral vectors.

**Fig. 5:**
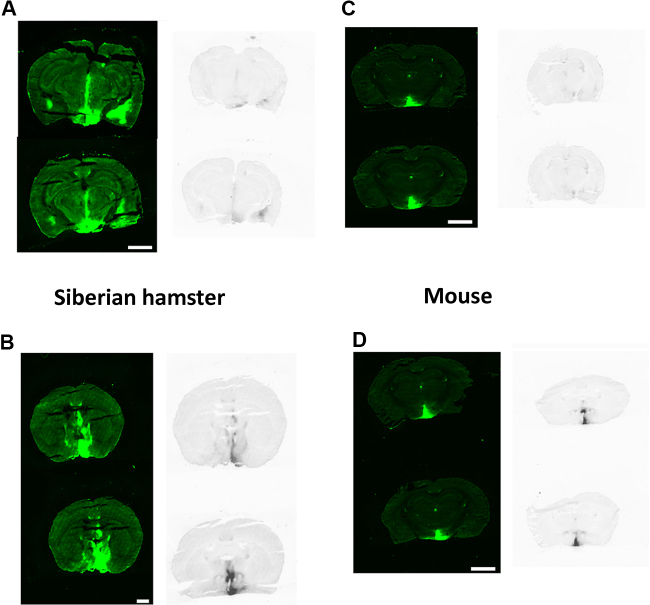
*VGF mRNA and eGFP expression patterns are similar following rAAV-VGF infusion into the hypothalamus of both Siberian hamsters and mice*. Representative pictures of eGFP expression (left) and VGF mRNA expression *via in situ* hybridisation (right) in the hypothalamus of Siberian hamsters (A and B) and mice (C and D) receiving bilateral injections of either rAAV-GFP (A and C) or rAAV-VGF (B and D) directed to the PVN. Scale bar = 2.5 mm.

*In situ* hybridisation analysis of the brain sections demonstrated over expression of VGF in the hypothalamus of all animals infused with rAAV-VGF vector (Fig. 5). This over-expression corresponded to the pattern of eGFP expression and associated fluorescence (Fig. 5). In contrast, animals infused with rAAV-GFP only expressed VGF mRNA in low amounts in the ARC (Fig. 5), indicating areas of endogenous VGF expression.

## 4 Discussion

The ability to co-express two or more genes in the same cells is highly desirable, particularly for identification of transfected cells following viral transfection *in vivo*. We have shown that the viral 2A sequence allows the simultaneous expression of both GFP and the gene of interest (VGF) in the brains of a non-standard laboratory species, the Siberian hamster, which is comparable to the mouse.

The role of VGF was first highlighted by the phenotype of mice lacking the functional vgf (non-acronymic) gene (Hahm et al., 1999), which suggested an anabolic role for this gene. However we have previously shown that VGF mRNA expression in the Siberian hamster is regulated in relation to changes in its body weight cycle, with significantly higher hypothalamic expression in the winter catabolic state as compared to the summer anabolic state (Barrett et al., 2005). Subsequent functional studies using VGF-derived peptides in both mice and Siberian hamsters, have suggested a catabolic role (Bartolomucci et al., 2006, Jethwa et al., 2007, Lewis et al., 2015). The lack of antibodies towards this gene for the detection of specific derived peptides, as well as our use of a non-standard animal model, directed us to develop a novel strategy to over-express VGF in the hypothalamus of adult Siberian hamsters. We also wanted to visualise transfected cells using a reporter gene, as previously observed in our studies on the over-expression of AgRP (Jethwa et al., 2010).

While our previous studies have shown that over-expression of two genes using IRES is feasible (Jethwa et al., 2010), the size of the IRES and VGF gene alone would have acceded the capacity of the rAAV and possible leading to issues with regards to sufficient titre. Thus we designed a vector utilising the viral 2A sequence for simultaneous expression of VGF mRNA and eGFP. The viral 2A sequence has not only been shown to be reliable, but also causes no cytotoxic effects in transgenic mice (Trichas et al., 2008). The viral 2A sequence is particularly useful in vector strategies (AAV and retroviral vectors) where the capacity to incorporate foreign DNA is limited. Indeed, the viral 2A sequence has been shown to reduce the size of the viral vector, improving transfection efficiency and viral titre, and thus transduction efficacy, leading to increased expression of both the gene of interest and reporter (Szymczak and Vignali, 2005).

We demonstrate the functionality of our plasmid vector (pAAV-VGF-GFP), which utilises the viral 2A sequence, in SH-SY5Y cells. High levels of eGFP expression were observed 72 h following transient transfection of pAAV-VGF-GFP into undifferentiated cells, which was comparable to the commercially available plasmid, pZsGreen1-N1. The increase in eGFP expression was accompanied by a significant increase in VGF mRNA in pAAV-VGF-GFP transfected cells as compared to cells transfected with pZsGreen1-N1. Furthermore, differentiation of the cell line to a more neuronal-like phenotype with retinoic acid resulted in expression of eGFP beyond the cell body into the neurite projections.

The AAV vector (rAAV-VGF) was then produced from this plasmid vector and infused into the hypothalamus of Siberian hamsters and mice to confirm the effectiveness of the viral 2A sequence *in vivo*. We have shown that the over-expression was still present in both species at 32 weeks post microinjection, while *in situ* hybridisation showed that the VGF mRNA expression was co-localised with eGFP expression. Whether this over-expression is equal in both species remains to be determined; unfortunately the *in situ* hybridisation method using the DIG does not lend itself to quantification, as the colour reaction is repeated to increase the intensity of the signal and reduce the background. This may account for the differences between the species, as sections were analysed in batches according to species rather than treatment.

Nonetheless, the function of viral 2A sequence appears not to show any attenuation in expression levels across generations (Trichas et al., 2008). Post mortem analysis revealed a high number of cells, over a large area of the brain, expressed the vectors at 32 weeks post infusion. Despite the bilateral infusion of 200 nl and a micropipette tip of 30 μm, it was apparent that cells 300–400 μm away also expressed the vector. Cells more dorsal in the brain adjacent to the tract of the micropipette also expressed the vectors. Higher lateral expression was noted in some of the animals, a reflection of a technical issue with the infusion of viral vectors at low volumes through small diameters, something we have noted previously (Jethwa et al., 2010). Furthermore this may result in areas of uneven expression potentially giving rise to differences between species and/or animals.

Thus, we have shown that the viral 2A sequence can be used successfully to over-express two genes simultaneously *in vitro* and *in vivo*. Indeed, Trichas et al. (2008) stated that the advantage of using 2A sequences over the commonly used IRES sequence is the ability to co-express both genes at nearly equal levels. Indeed 10 fold differences in IRES-controlled expression have been noted (Martin et al., 2006), while the length of the gene preceding (5′) the IRES has recently been shown to influence the expression of the following (3′ to the IRES) reporter (Payne et al., 2013), demonstrating the unreliability of the sequence for simultaneous gene expression. These effects have not been observed for plasmid and/or viral vectors which utilise the viral 2A sequence, which is particularly important when high levels of expression of both genes are required (Furler et al., 2001). However, as with all systems, there is a potential limitation to the use of the viral 2A sequence. Since the viral 2A sequence is added to the C-terminal of the protein encoded upstream of the sequence, this may affect function and/or activity of that protein, although this has not been observed in any of the studies published to date (Furler et al., 2001, Trichas et al., 2008). Further studies concerning the function and safety of the viral 2A sequence are required, but no adverse effects were noted in our studies following infusion of viral 2A sequence containing vectors. Indeed, the sequence successfully mediated co-translational ‘cleavage’ to express two genes within the hypothalamus of Siberian hamsters akin to that in the mouse and other rodent models (Furler et al., 2001, Trichas et al., 2008).

Overall, the results further support the use of AAV vectors for manipulating gene function *in vivo*, particularly in non-standard laboratory models. We have shown that the AAV construct using the viral 2A sequence resulted in simultaneous expression of VGF and eGFP, both *in vitro* and *in vivo*. Furthermore, the AAV construct results in long-term expression *in vivo*.

## Figures and Tables

**Table 1:** PCR primers used for the synthesis of VGF-2A fragment, to determine endogenous VGFmRNA and cyclophilin control.

Gene	Forward primer (5′–3′)	Reverse primer (5′–3′)
Modified PCR primers (for amplification of VGF cDNA)	CCC GGG AAG CTT **ACC ATG** AAA ACC TTC ACG TTG CCG GCA TCC	GGG CCC TGG GCC AGG ATT CTC CTC GAC GTC ACC GCA TGT TAG CAG ACT TCC TCT GCC CTC TCC ACT GCCGAG CAC GTG CTG CTG CAC CGC CCG
VGF mRNA	GAC CCT CCT CTC CAC CTC TC	ACC GGC TCT TTA TGC TCA GA
Cyclophilin A	TCC TGC TTT CAA GAA TTA TTC C	ATT CGA GTT GTC ACA GTC AGC

The modified PCR primers for the amplification of VGF cDNA (Accession no.: NM_001039385.1) from the pSC-B-AMP/KAN blunt cloning vector to produce the VGF-2A fragment. The forward primer contains a Kozak sequence (in bold) for initiation of translation, while the reverse primer contains the viral 2A sequence (underlined) and a point mutation to allow the removal of the stop codon from the VGF cDNA. Both primers contained unique restriction sites to aid the sub-cloning process. The VGF mRNA and cyclophilin primers were designed from database sequences. Sequence data was input into Primer3 in FASTA format and primers were designed using the default criteria (Primer size; 18–27 bp, Melting temperature 55–62 °C, Amplicon size 50–150 bp) and obtained from Sigma, Dorset, UK.

**Table 2:** Comparison of food intake and locomotor activity one week post viral infusion in the Siberian hamster and mouse.

	Food intake (g)		Locomotor activity (beam breaks)	
	rAAV-GFP	rAAV-VGF	rAAV-GFP	rAAV-VGF
Siberian hamster	3.71 ± 0.24	4.01 ± 0.07	23.10 ± 5.10	23.11 ± 7.38
Mouse	3.76 ± 0.32	4.13 ± 0.25	97.93 ± 9.17	100.13 ± 17.16

Mean food intake (g) measured in the home cage and mean 24 h values for the number of beam breaks in the metabolic cages (Comprehensive Lab Animal Monitoring System; CLAMS). Values are mean ± SEM.
